# Measuring Climate Change Knowledge, Attitudes, Behaviours and Activism Among Older Adults

**DOI:** 10.1093/geroni/igaf122.1267

**Published:** 2025-12-31

**Authors:** Paola Zaninotto, Matthew Prina

**Affiliations:** University College London, London, England, United Kingdom; Newcastle University, Newcastle upon Tyne, England, United Kingdom

## Abstract

Older adults are particularly vulnerable to climate-related hazards such as extreme heat, flooding, and severe storms, yet their perspectives and contributions to climate resilience remain underrepresented in research. We highlight the importance of incorporating climate-related themes into ageing studies and propose a set of short, validated questions to assess older adults’ knowledge, concerns, preparedness, behaviours, and involvement in climate action. These questions have undergone cognitive testing with diverse participants to ensure clarity, accessibility, and relevance. Expert review helped refine content, simplifying wording, response scales, and framing to better capture lifetime actions rather than limited timeframes. Designed for flexibility across research contexts, they are easy to implement via face-to-face interviews, telephone, or self-completion. By integrating these measures, ageing research can provide valuable insights to inform policies and initiatives that reduce climate risks while promoting sustainable and healthy ageing. Recognising older adults’ voices and capacities is crucial in fostering climate resilience.

